# Artificial intelligence assists identification and pathologic classification of glomerular lesions in patients with diabetic nephropathy

**DOI:** 10.1186/s12967-024-05221-8

**Published:** 2024-04-29

**Authors:** Qunjuan Lei, Xiaoshuai Hou, Xumeng Liu, Dongmei Liang, Yun Fan, Feng Xu, Shaoshan Liang, Dandan Liang, Jing Yang, Guotong Xie, Zhihong Liu, Caihong Zeng

**Affiliations:** 1 National Clinical Research Center of Kidney Diseases, Jinling Hospital, Nanjing University School of Medicine, 305 East Zhongshan Road, Nanjing, 210009 China; 2 Ping An Healthcare Technology, 206 Kaibin Road, Shanghai, 200030 China; 3 Ping An Healthcare and Technology Company Limited, Shanghai, China; 4 Ping An International Smart City Technology Co., Shanghai, China

**Keywords:** Artificial intelligence, Diabetic nephropathy, Glomerulus, Pathology, Identification

## Abstract

**Background:**

Glomerular lesions are the main injuries of diabetic nephropathy (DN) and are used as a crucial index for pathologic classification. Manual quantification of these morphologic features currently used is semi-quantitative and time-consuming. Automatically quantifying glomerular morphologic features is urgently needed.

**Methods:**

A series of convolutional neural networks (CNN) were designed to identify and classify glomerular morphologic features in DN patients. Associations of these digital features with pathologic classification and prognosis were further analyzed.

**Results:**

Our CNN-based model achieved a 0.928 F1-score for global glomerulosclerosis and 0.953 F1-score for Kimmelstiel-Wilson lesion, further obtained a dice of 0.870 for the mesangial area and F1-score beyond 0.839 for three glomerular intrinsic cells. As the pathologic classes increased, mesangial cell numbers and mesangial area increased, and podocyte numbers decreased (p for all < 0.001), while endothelial cell numbers remained stable (p = 0.431). Glomeruli with Kimmelstiel-Wilson lesion showed more severe podocyte deletion compared to those without (p < 0.001). Furthermore, CNN-based classifications showed moderate agreement with pathologists-based classification, the kappa value between the CNN model 3 and pathologists reached 0.624 (ranging from 0.529 to 0.688, p < 0.001). Notably, CNN-based classifications obtained equivalent performance to pathologists-based classifications on predicting baseline and long-term renal function.

**Conclusion:**

Our CNN-based model is promising in assisting the identification and pathologic classification of glomerular lesions in DN patients.

**Supplementary Information:**

The online version contains supplementary material available at 10.1186/s12967-024-05221-8.

## Introduction

Diabetic nephropathy (DN) is one of the main causes of chronic kidney disease worldwide [1]. Patients with DN are characterized by continuously progressive proteinuria and renal function decline, and most of them are diagnosed with clinical characteristics without undergoing renal biopsy. However, previous studies have reported 3% to 82.9% incidence of nondiabetic renal diseases among patients with diabetes [2]. Recently, renal biopsy has still been the gold standard for clarifying injury patterns among patients with diabetes.

The glomerular lesion, the main pathological feature of DN [3, 4], covers a spectrum of lesion manifestations, mainly involving mesangial cell proliferation [5, 6], mesangial matrix accumulation [3, 4], Kimmelstiel-Wilson lesion [4], and podocyte depletion [7–9]. Currently, a widely used standard for pathologic classification, proposed by the Renal Pathology Society (RPS) in 2010, is based on the evaluation of above glomerular morphological features [4] and has been verified to show significant prognostic value among patients with DN [10]. In clinical practice, these morphological features are evaluated visually by pathologists using qualitative or semi-quantitative methods. For intraglomerular features (e.g., mesangial region, intrinsic cells), several complicated methods such as Weibel and Gomez method, disector/fractionator combination, and Wiggins method were reported to quantify them [11, 12]. However, these methods require extra procedures for slide preparations (e.g., special staining procedure) and manual counting, which is time-consuming in the clinical setting. There need for a more rapid or automatic method to quantify glomerular morphological features just using routine-stained sections to assist pathologist’s routine-work.

Deep learning paves the way for a paradigm shift from descriptive to quantitative pathology and has the potential to augment renal pathologists [13, 14]. Convolutional neural networks (CNN), one of the most popular deep learning algorithms at present, have outperformed other deep learning algorithms in automatic image analysis [15, 16]. In the field of renal pathology, deep learning algorithms have been applied successfully to identify glomeruli, tubules, and interstitium in digital slides from transplant biopsies [17] or patients with Minimal Change Disease [18]. However, these studies didn’t further recognize the intraglomerular features and just only used relatively normal and lightly damaged tissue. Ginley et al. [19] developed a machine learning method to extract intraglomerular features compounded by color, texture, and nucleus distance in patients with diabetic kidney disease. Those features were used to train a deep learning network to classify glomerular lesions which obtained moderate agreement with pathologists. However, those features couldn’t directly correspond to any actual glomerular compartments or lesions, making it relatively difficult to explain.

Our center has established an analytic renal pathology system (ARPS, integrated by multiple well-trained networks) to automatically detect glomeruli types and intrinsic cells by utilizing routine PAS-stained sections from IgA nephropathy (IgAN) patients [20]. This study aimed to design the CNN-based model to automatically quantify actual glomerular morphological features in a large sample of DN patients, including the types of glomerular lesions and intraglomerular features. The quantified glomerular features will be used to assist pathologic classification and prognosis evaluation.

## Methods

This retrospective study consists of four main parts as follows: (i) training CNN model to identify glomeruli types and intraglomerular features, (ii) characterizing glomerular morphological features, (iii) clinical application 1: CNN-based-pathologic classification versus pathologists-based classification, (iv) clinical application 2: associations of CNN-based classes with baseline and long-term renal function versus that of pathologists-based classes. This study follows the principles of the Helsinki Declaration and was approved by the Ethics Committee of Jinling Hospital (2019NZKYKS-008-01).

### Identifying glomerular morphological features

#### Digital slides preparation

The detailed patient’s clinical information was described in Additional file 1: Supplementary Method I. All patients have undergone a percutaneous renal puncture biopsy under ultrasound. Biopsy tissue specimens for light microscopy were fixed in formalin and embedded in paraffin. Slices (2-μm thickness) were routinely stained with hematoxylin–eosin, periodic acid–Schiff (PAS), periodic acid-silver methenamine, and Masson’s trichrome. Archival PAS-stained slides were scanned by Aperio’s ScanScope AT Turbo Scanner (Leica, Wetzlar, Germany) under 40× magnification at a resolution of 0.25 μm/pixel. Those whole slide images (WSIs) having obvious compression artifacts or decolorization were excluded for quality control.

#### CNN design

**Fig. 1:**
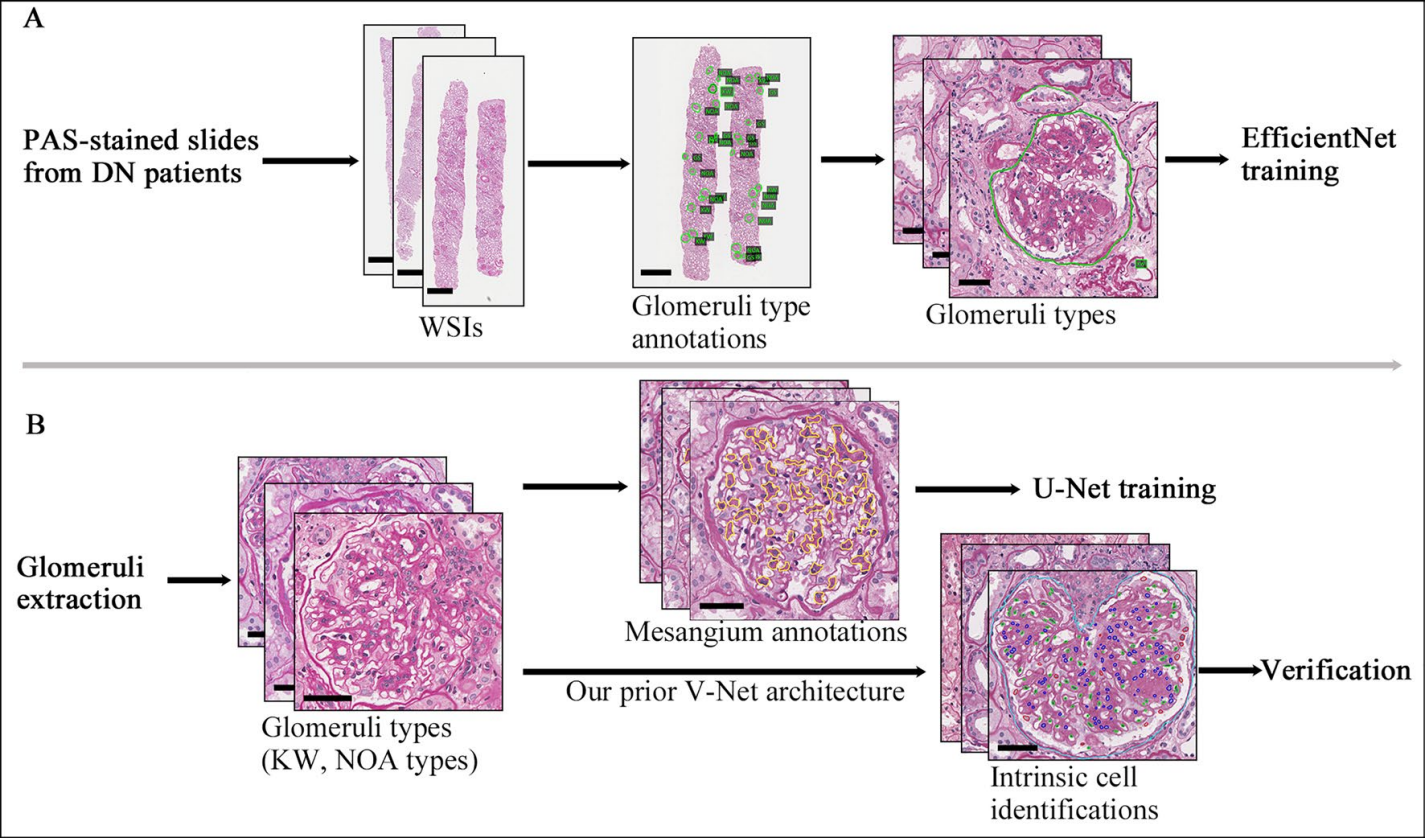
Schematic illustration of our CNN model for identifying glomerular morphological features. **A** The training procedure of EfficientNet architecture for identifying different glomeruli types. **B** The training procedure of U-Net architecture for segmenting mesangial area and the verification procedure of our prior V-Net architecture for identifying three glomerular intrinsic cells. Scale bars mean 750 μm in WSIs and 50 μm in single glomerulus images

To construct a CNN architecture that can appropriately categorize glomeruli types in patients with DN, the following subcategories of glomeruli were applied: global glomerulosclerosis (GS), segmental glomerulosclerosis (SS), crescent (C), Kimmelstiel-Wilson lesion (KW), and none of the above lesions (NOA). Regions excluding glomeruli were defined as negative samples (Neg). The glomeruli on PAS-stained WSI slides were annotated by an experienced junior pathologist (who has at least 3 years experience in renal pathology) using Aperio’s ImageScope software through labeling along the margin of Bowman capsule and tagging on their subcategories. To improve the model performance on identifying KW lesions for further pathologic classification, when a glomerulus had KW lesions accompanied by another lesion such as SS or C lesion, it was still annotated as a KW glomerulus. These annotations were checked by three senior pathologists (who all have more than 10 years experience, signed about 1600 renal biopsy reports per year, and participated in the study of the Oxford Classification of IgAN [21, 22]). The above WSI images and annotation information were utilized to train the CNN model. The detailed CNN training procedure is described in Additional file 1: Supplementary Methods II. Figure 1 depicts the detailed training process of the above CNN models. The testing data were used for evaluating the above classification models (glomeruli types identification and intrinsic cells prediction) and segmenting model (mesangial area segmentation). The detailed evaluation procedures were described in Additional file 1: Supplementary Methods III. Our CNN Model is developed on PyCharm Community v2021.1.1 and PyTorch v1.8.1 platform. The CNN algorithms were uploaded to the GitHub website (https://github.com/xavierhou/kidney_pathology_AI).

### Characterizing glomerular morphological features

The above well-trained CNN networks were used to identify glomeruli types and further quantify intraglomerular features (mesangial area and three intrinsic cells) in a large sample of DN patients. The percent glomeruli types were calculated to reveal the involvement extent of such type in one patient. The detailed calculating methods for intraglomerular features in one patient were described in Additional file 1: Supplementary Methods IV. The glomerular lesions were classified by three senior pathologists using the standard proposed by RPS in 2010 [4].

### CNN-based classification versus pathologists-based classification

Based on the above glomeruli types and intraglomerular features quantified by our CNN methods, we tried to classify glomerular lesions according to the RPS standard. Firstly, patients with percent GS > 50% were picked out as class IV. Patients with the presence of KW lesions and with percent GS ≤ 50% were assigned to class III. Then, the receiver operating characteristic (ROC) curve of each measured intraglomerular morphometric for pathologists-based classes was performed. The area under the ROC curve (AUC) was used to evaluate the predictive capacity of each morphometric on classifying the remaining patients (not satisfying class IV and class III). The optimal cutoff value of the intraglomerular morphometric with a larger AUC value was used to assign patients from class I to class IIb. Furthermore, Cohen’s kappa value was used to evaluate the consistency between CNN-based classification and that by senior pathologists.

### Associations of CNN-based classification with baseline clinical indicators and long-term prognosis

Patients were performed follow-up commonly at 3- to 6-month intervals after biopsy which adjusted according to their specific conditions. The follow-up period was defined as the time from renal biopsy to the last follow-up visit (by October 30, 2021). Prognostic analyses were conducted among these patients with follow-up periods ≥ 1 month and baseline eGFR ≥ 30 ml/min/1.73 m^2^. We collected the serial measurements of serum creatinine and proteinuria from each visit after biopsy until the event of ESRD or the last follow-up. The slope of eGFR calculated by the principal of least squares using linear regression was used to reveal the rate of renal function decline per year after biopsy. We used time-average proteinuria (TA-P) to reflect the average proteinuria level after biopsy, which was defined as the ratio of the area under the curve of serial proteinuria measurements to the follow-up period [23]. The event of ESRD was defined as eGFR < 15 mL/min/1.73 m^2^ or initiation of dialysis over 3 months, or renal transplantation. Spearman correlation was performed to estimate the associations of CNN-based classes or pathologist-based classes with baseline clinical indicators and long-term renal function.

### Statistical analyses

Continuous variables were presented as median (interquartile range: IQR), and inter-group comparisons were performed by Mann Whitney U test or Kruskal–Wallis H test as appropriate. Categorical variables were presented as numbers (percentages). The r coefficients were compared for significance using the Z-score method [24, 25]. The data were analyzed using SPSS version 25.0 and plotted in GraphPad Prim 8. Two-sided P values were reported, and P < 0.05 was statistically significant.

## Results

### Patients’ characteristics

A total of 631 patients with biopsy-proven DN were enrolled. The enrolled patients were allocated to different subsets for different tasks. The internal application subset was from cases involving in glomeruli types model or mesangial area model, and additional cases (not involved in any model training) were used as the external application subset. The detailed clinical characteristics of different subsets are described in Additional file 1: Table S1.

### Identifying glomerular morphological features

#### Glomeruli types and mesangial area

A confusion matrix displayed the prediction of glomeruli types by our CNN model relative to the ground truth by pathologists (Additional file 1: Table S2). Our CNN model achieved excellent performance in identifying KW and GS glomeruli, with F1-scores of 0.953 and 0.928, respectively (Table 1). Next, we developed another CNN algorithm for segmenting the mesangial region in these two types of glomerulus. The average dice for segmenting mesangial area was 0.870, and the dice for NOA and KW glomeruli were 0.864 and 0.884, respectively.

**Table 1:** Performance of our CNN model on identifying different glomeruli types

Glomeruli types	Accuracy	Specificity	Precision	Recall	F1-score
Global glomerulosclerosis	0.984	0.985	0.881	0.981	0.928
Segmental glomerulosclerosis	0.956	0.982	0.692	0.595	0.640
Kimmelstiel–Wilson lesions	0.988	0.997	0.979	0.929	0.953
None of the above	0.947	0.944	0.914	0.952	0.933

#### Glomerular intrinsic cells

**Fig. 2:**
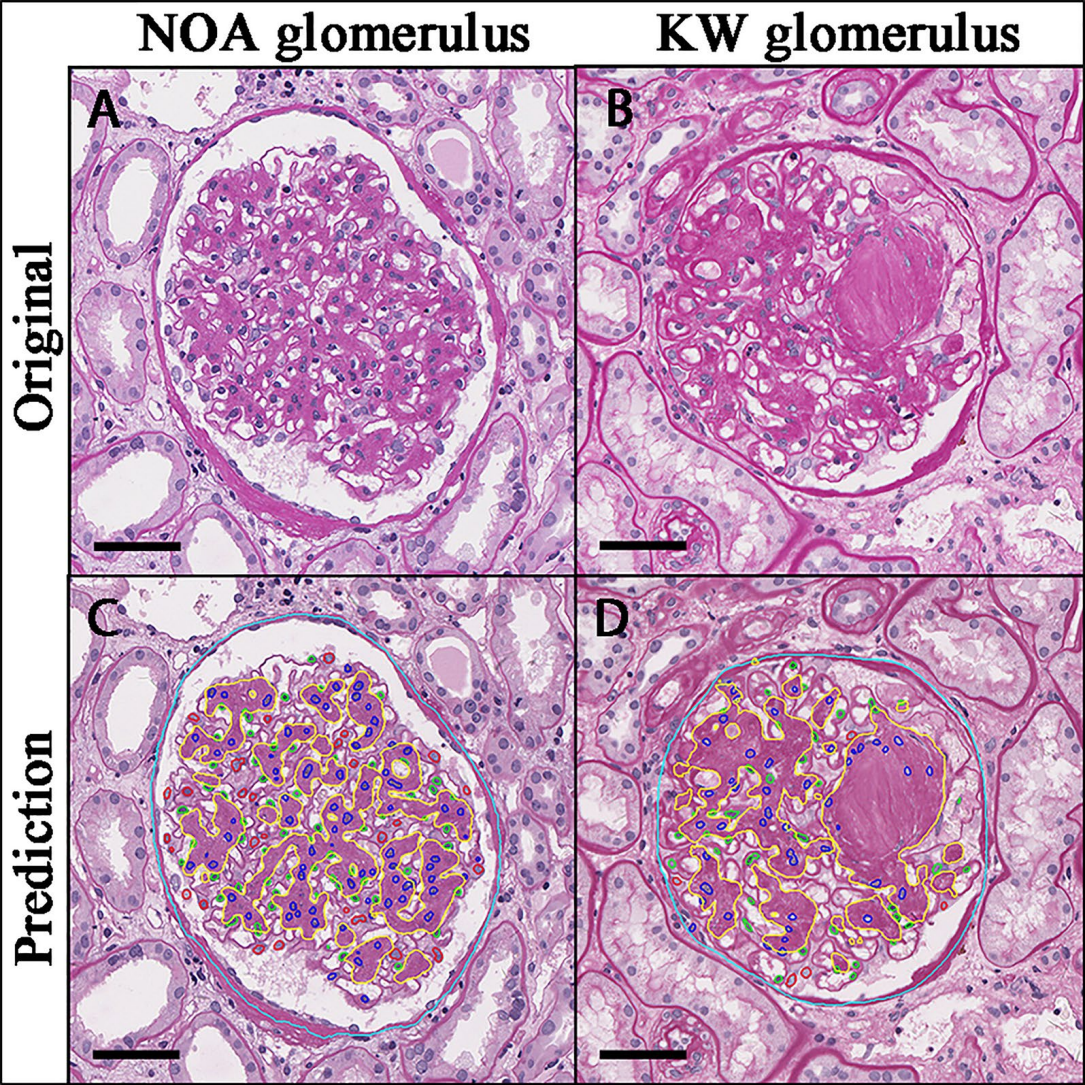
Our CNN architectures identify intraglomerular features. Original images derived directly from WSI slides (**A** NOA glomerulus, **B** KW glomerulus). Prediction images (**C**, **D**) describe the predicted results of intraglomerular features from the original images (cyan: Bowman capsules, red: podocytes, blue: mesangial cells, green: endothelial cells, yellow: mesangial regions). Scale bar: 50 μm

In view of the good performance of our previous ARPS for the recognition of glomerular intrinsic cells in IgA patients, we firstly verified the performance of the ARPS system on identifying glomerular intrinsic cells in DN patients, a total of 13,072 intraglomerular cells originating from 50 NOA glomeruli and 50 KW glomeruli were selected. The confusion matrix of intrinsic cells is described in Additional file 1: Table S3. This model achieved F1-scores beyond 0.839 in predicting three intrinsic cells in DN patients, obtaining the highest F1-score in predicting mesangial cells, followed by endothelial cells and podocytes (Table 2). Figure 2 presents the results of segmenting mesangial area and predicting three intrinsic cells by our CNN models.

**Table 2:** Performance of the V-Net architecture from the ARPS system on identifying three glomerular intrinsic cells in patients with DN

Intrinsic cells	Accuracy	Specificity	Precision	Recall	F1-score
Mesangial cells	0.942	0.953	0.932	0.926	0.929
Endothelial cells	0.930	0.970	0.939	0.855	0.895
Podocytes	0.961	0.986	0.890	0.793	0.839

### Characterizing glomerular morphological features

**Fig. 3:**
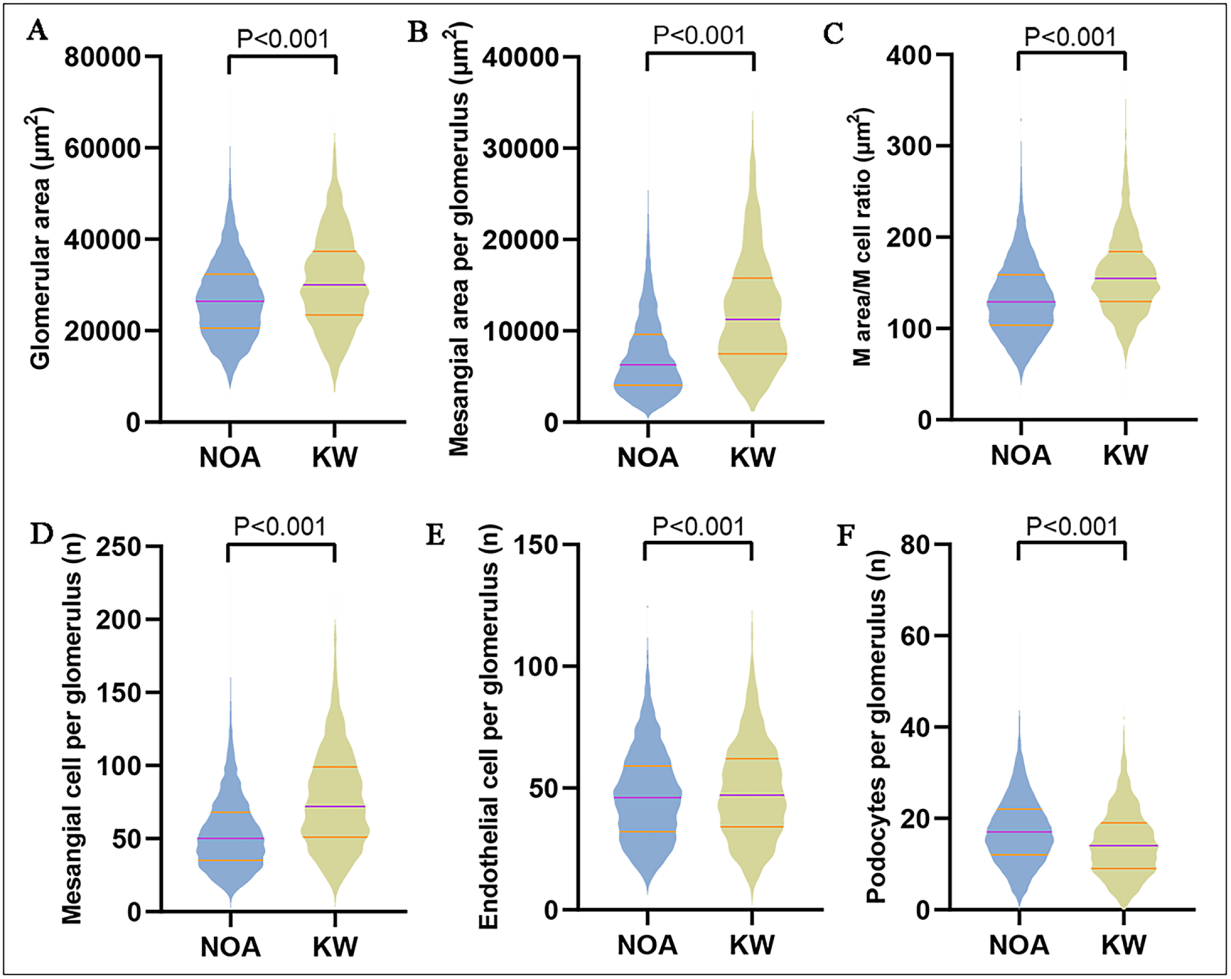
Violin plots depict the distribution of each intraglomerular feature between NOA and KW glomeruli from a pooled 11,188 midsection glomerular image (NOA: 8181, KW: 3007). M: mesangial, E: endothelial, P: podocytes. The purple line: the median value, the orange line: the interquartile range. Inter-group comparisons were performed by Mann Whitney U test

Then our CNN architecture was used to quantify these features in a large sample of DN patients. Of 340 cases, 21,180 glomeruli were identified by our CNN model in which the predicted percentages of different glomeruli types were 31.7% (GS), 3.9% (SS), 0.6% (C), 15.5% (KW), and 48.2% (NOA), respectively. 11,188 KW and NOA glomeruli in the midsection (total glomerular cells ≥ 50) were picked out for further intraglomerular features analysis. For patients’ level, as the pathologic classes increased, mesangial cell numbers, mesangial area, mesangial area fraction, and mesangial area/mesangial cell ratio increased significantly; podocyte numbers decreased significantly; while endothelial cell numbers remained relatively stable (Additional file 1: Table S4). In addition, we also calculated the changes in intrinsic cells relative to the glomerular area (termed cell density). It showed similar change trends to the above absolute results (Additional file 1: Table S5). For the glomerular level, we compared the intraglomerular morphometrics between NOA and KW glomeruli. KW glomeruli showed significantly increased glomerular area, mesangial area, mesangial cell numbers, mesangial area/mesangial cell ratio, and endothelial cell numbers, but severe podocytes depletion compared to NOA glomeruli (Fig. 3). In addition, KW glomeruli also showed a significantly increased ratio of mesangial cells and endothelial cells, while decreased podocyte ratio compared to NOA glomeruli (Additional file 1: Table S6).

**Fig. 4:**
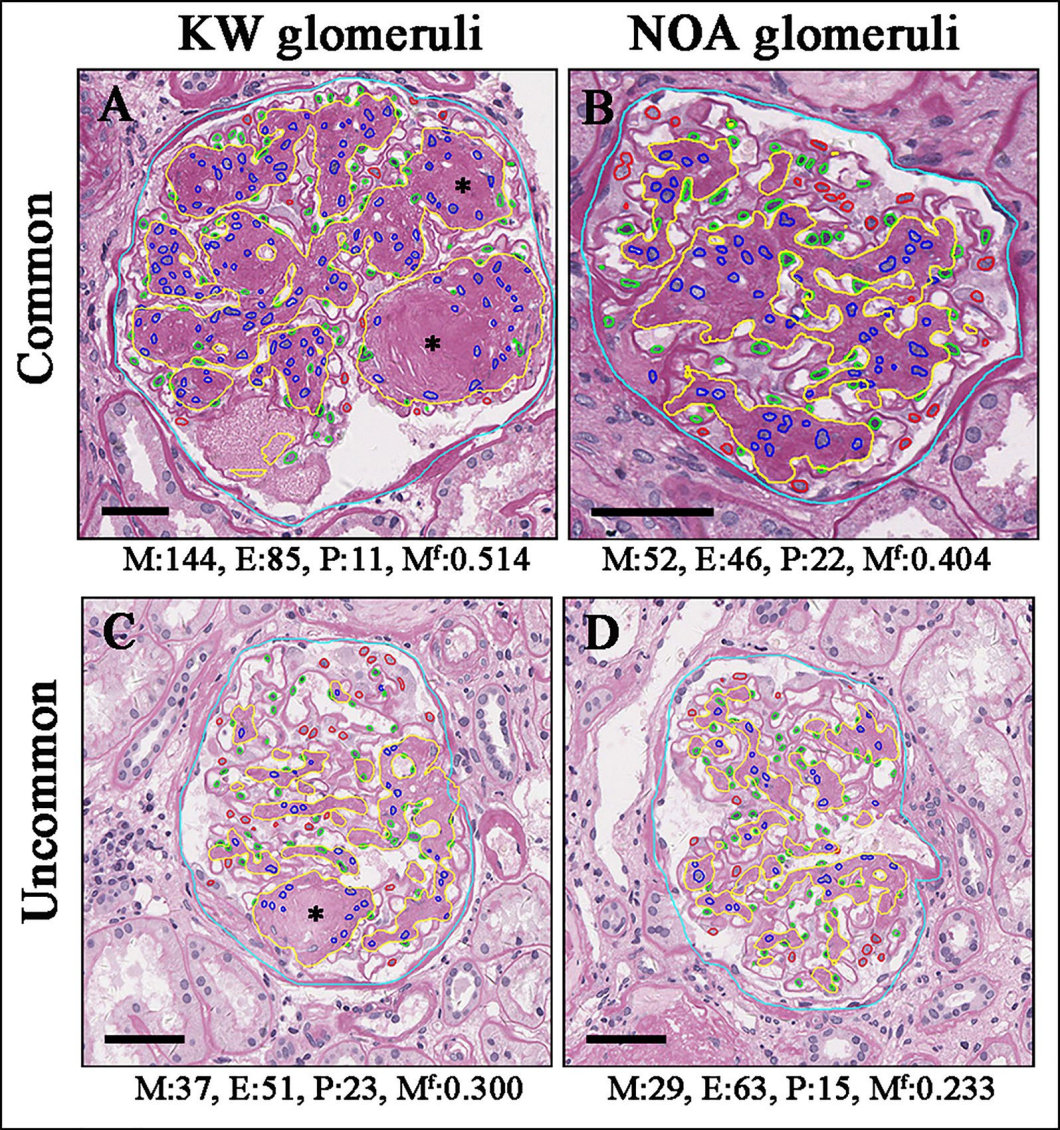
Intraglomerular lesions within one patient are not parallel to each other. The common presentation showed that a patient in class III had Kimmelstiel–Wilson (KW) lesions (**A**) accompanied by severe mesangial expansion (**B**). While in uncommon presentation, a patient in class III had a KW lesion (**C**) accompanied by mild mesangial expansion (**D**). Cyan: Bowman capsule; red: podocytes (P); blue: mesangial cells (M); green: endothelial cells (E); yellow: mesangial area; asterisk, KW lesions. M^f^: mesangial area fraction. Scale bar: 50 μm

Except for most patients presenting strong correlations of glomerular feature levels with their pathologic classes, we also observed a small subset of DN patients who fulfilled the criteria of class III or class IV but presented with relatively mild mesangial expansion. If we defined the value of mesangial area fraction < 95% of that in ≤ class IIa as mild mesangial expansion (mesangial area fraction < 0.2596). A total of 24 (9.7%) cases in class III or class IV have mild mesangial expansion (Fig. 4). It indicated that the severity of a lesion in one glomerulus could be not always parallel to that in another within the same patient.

### CNN-identified glomerular morphological features determine pathologic classification

#### Evaluating the effect of individual intraglomerular morphometric on predicting early pathologic classes

The early pathological stage (from class I to class IIb) is divided by the width of the mesangial region according to the RPS classification system. Here, ROC curves were conducted to evaluate the effect of each morphometric on distinguishing between early pathologic classes using the internal subset. It showed that all significant features were involved in mesangium (Additional file 1: Table S7). The top three features comprised average mesangial area, mesangial area fraction, and mesangial area/mesangial cell ratio, with the AUCs beyond 0.809. The optimal cutoff values of these mesangium-related indices were used to classify patients from class I to class IIb respectively.

#### CNN-based versus pathologist-based classifications

After the glomerular features in WSIs were quantified by our CNN algorithms, the percent GS > 50% and the presence of KW lesions were used to screen out patients in class IV and class III, respectively. Then, the optimal cutoff values from the top three mesangium-related features were used to distinguish between early classes respectively. As shown in Table 3, model 3 (combined with mesangial area fraction) obtained the highest Cohen’s kappa value with pathologists on predicting pathologic classes in the internal subset. It reached 0.624 (CNN-pathologist pair ranged from 0.529 to 0.688), achieving moderate agreement. In the external subset, model 1 (combined with average mesangial area) obtained the highest Cohen’s kappa of 0.663 (CNN-pathologist pair from 0.655 to 0.699). Additional file 1: Fig. S1 describes the detailed procedure to classify glomerular lesions in DN patients. We also assessed the Cohen’s kappa values between three experienced pathologists in 65 cases, which are 0.773 (pathologist 1-pathologist 2), 0.797 (pathologist 1-pathologist 3), and 0.754 (pathologist 2-pathologist 3), respectively.

**Table 3:** The consistency between the CNN-based and pathologist-based pathologic classes

Cohen’s kappa value	CNN-based classes					
	Internal application subset			External application subset		
	Model 1	Model 2	Model 3	Model 1	Model 2	Model 3
Pathologist 1	0.475	0.457	0.529	0.667	0.585	0.640
Pathologist 2	0.688	0.722	0.688	0.655	0.588	0.423
Pathologist 3	0.653	0.655	0.654	0.699	0.618	0.741
Total	0.604	0.587	0.624	0.663	0.592	0.602

Model 1 was the CNN-based classification derived from combination of percent GS, the presence of KW lesions with the optimal cutoff values of average mesangial area; Model 2 derived from combination of percent GS, the presence of KW lesions with the optimal cutoff values of mesangial area/mesangial cell ratio; Model 3 derived from combination of percent GS, the presence of KW lesions with the optimal cutoff values of mesangial area fraction

### CNN-based classification achieved equivalent performance to pathologist-based classification in predicting renal function

The trajectories of kidney function among our population were evaluated. Of 250 patients having follow-up information, 49 cases (19.6%) entered ESRD. A total of 3126 measures of eGFR (from 250 cases) and 1532 measures of proteinuria (from 248 cases), including the baseline measures, were collected. The average follow-up period was 2.58 years for eGFR and 2.52 years for proteinuria. The average times of measurements in one patient were 11.9 for eGFR and 5.9 for proteinuria. The median (IQR) eGFR slope of different classes by pathologists was − 1.89 (− 3.26, 0.71) mL/min/1.73 m^2^/year (class I), − 1.65 (− 7.60, 1.23) mL/min/1.73 m^2^/year (class IIa), − 3.34 (− 7.05, − 3.33) mL/min/1.73 m^2^/year (class IIb), − 11.60 (− 21.46, − 4.61) mL/min/1.73 m^2^/year (class III), and − 13.75 (− 22.73, − 7.58) mL/min/1.73 m^2^/year (class IV), respectively. The median (IQR) TA-P levels of different classes were 0.73 (0.48, 1.05) g/24 h (class I), 1.41 (0.71, 2.18) g/24 h (class IIa), 1.60 (0.66, 3.26) g/24 h (class IIb), 3.92 (2.28, 6.51) g/24 h (class III), and 5.08 (2.73, 7.69) g/24 h (class IV), respectively.

Firstly, we analyzed the CNN-based classes derived from model 1 (combined with the average mesangial area) in the internal subset. It was associated significantly with the baseline proteinuria and eGFR level, TA-P level, and eGFR slope after biopsy, as well as the event of ESRD. The Spearman r values of the classes from model 1 with these clinical prognostic indicators were comparable to that of pathologist-based classes with these indicators using the Z-scores method (Table 4). It was consistent with the results in the external subset (Additional file 1: Table S8). Model 2 (combining with mesangial area/mesangial cell ratio) and model 3 (combining with mesangial area fraction) also showed similar results (Additional file 1: Tables S9–S12). The above results indicated that CNN-based classifications performed equally to pathologist-based classifications on predicting baseline and long-term renal function.

**Table 4:** Spearman correlation of clinical prognostic indicators with CNN-based or pathologists-based classes in the internal application subset

Indicators	Proteinuria				eGFR					
	Proteinuria at biopsy, g/24h (n = 226)		Time-average proteinuria, g/24h (n = 157)		eGFR at biopsy, ml/min/1.73 m^2^ (n = 226)		eGFR slope, ml/min/1.73 m^2^/year (n = 157)		Event of ESRD (n = 157)	
Spearman correlation (*r*, *P *value)	*r*	*P *value	*r*	*P *value	*r*	*P *value	*r*	*P *value	*r*	*P *value
Pathologists-based classes	0.327	< 0.001	0.447	< 0.001	− 0.443	< 0.001	− 0.363	< 0.001	0.250	0.002
CNN-based classes	0.368	< 0.001	0.384	< 0.001	− 0.371	< 0.001	− 0.318	< 0.001	0.229	0.004

Z-scores (Z,* P *value)	Z	*P *value	Z	*P *value	Z	*P *value	Z	*P *value	Z	*P *value
CNN vs pathologists	− 0.878	0.380	1.162	0.245	− 1.591	0.112	0.798	0.425	0.359	0.720

CNN-based classes derived from Model 1 (combined with average mesangial area). The Z-score method was used to test the difference between the r coefficients from pathologists-based classes and those from CNN-based classes. The Spearman r coefficient of CNN-based classes with pathologists-based classes was 0.718. The results of the Z-score method were given from hittner2003

## Discussion

Glomerular lesions were proved to best reflect the natural course of progressive DN and were used for pathologic classification [4, 26, 27]. In this study, we constructed a CNN-based method to automatically characterize glomerular morphological features from the specimen sections in a large sample of DN patients. Several intraglomerular features showed strong correlations with the pathologic classes, including mesangial area, numbers of mesangial cells, and podocytes. Furthermore, the performance of CNN-based classification was equivalent to that of pathologist-based classification in predicting baseline renal function and prognosis. It remained robust in the external dataset.

The glomerular lesion develops as a continuous morphologic spectrum. Quantification of glomerular lesions is of great significance for pathologic classification and prognosis evaluation in DN patients. In clinical practice, quantifications of intraglomerular features are unscalable and impractical for pathologists in routine pathology workflow [13]. Our study developed a CNN method to identify glomeruli types, segment mesangial area, as well as recognize and count three intrinsic cells automatically and accurately just based on routine PAS-stained slides. To our knowledge, this is the first time to simultaneously identify these key morphological features using CNN algorithms among patients with DN. As the pathologic classes increased, the number of mesangial cells and mesangial area increased significantly, endothelial cell number remained stable; while podocyte number decreased gradually. The current pathologic classification system assigns diverse patients into several given categories based on partial injury characteristics. It reduced the ability to capture the heterogeneity of histologic lesions. Notably, through using our CNN models, we observed an uncommon pathological presentation that patients in class III and class IV could have mild mesangial expansion in a proportion of glomeruli, indicating heterogeneous glomerular lesions among patients even within the same pathologic class.

The pathologic classification proposed by RPS [4] was verified to be significantly associated with renal outcomes in DN patients [10]. However, this classification system was a manual assessment and labor-intensive. In this study, mesangial area-related features were found to obtain superior performance than other features in distinguishing patients in early classes. It was consistent with the visual estimation based on mesangial width by pathologists [4]. The combination of these superior mesangial features with percent GS and KW lesions was further used for pathologic classification, achieving moderate agreement with pathologists-based classes. The external subset showed similar agreement, indicating the strong robustness and generalization ability of our CNN models in pathological classification.

Pathologic classification was also used to reveal the prognosis of patients. Our CNN-based classification achieved identical performance to pathologists-based classification for predicting the baseline and long-term renal function (including proteinuria and eGFR), though only achieving moderate agreement with pathologists. Additionally, in the external subset, CNN-based classes had a slightly higher correlation coefficient (although not statistically) with renal function indices compared to pathologists-based classes. It may be due to the relatively mild lesions in the external subset.

However, this study also has certain limitations. Firstly, we only measured intraglomerular morphological features in a substantial proportion of glomeruli such as KW lesions or none of the above lesions. The changes in intraglomerular morphometrics in a small subset of glomeruli with segment glomerulosclerosis are still unknown. Secondly, although we tried our best to diminish the bias from the glomerular area and non-midsections, the quantifications of glomerular features in this study need to be further verified by other morphological methods. Importantly, this is a single-center study, lacking external validation in other centers. We are planning to evaluate the effectiveness of our CNN models in other centers.

The CNN algorithms can be trained to automatically quantify the glomerular and intraglomerular morphological features on whole-slide images just using routine-stained slides from patients with DN. Furthermore, the quantified glomerular features derived from the well-trained CNN model hold large potential to assist pathologic classification and prognosis evaluation in clinical practice, hence enhancing precision medicine in DN patients.

### Supplementary Information

**Additional file 1.** Supplementary Material

## Data Availability

The data underlying this article will be shared on reasonable request to the corresponding author.
