# Supplementary material for: Artificial intelligence assists identification and pathologic classification of glomerular lesions in patients with diabetic nephropathy
Source: J Transl Med. 2024 Apr 29;22:397. doi: 10.1186/s12967-024-05221-8 (PMC11059590; doi:10.1186/s12967-024-05221-8)

**Supplementary Methods**

**I. Patients’ clinical features**

Adult patients who underwent renal biopsy at the National Clinical Research Center of Kidney Diseases from 2003 to 2019 were retrospectively reviewed and selected. Inclusion criteria were as follows: (i) age ≥18 years; (ii) type 2 diabetes; (iii) DN diagnosed by renal biopsy^1^. All patients were assessed by light, immunofluorescence, and electron microscopy. Patients with transplanted renal diabetic nephropathy or combined with other kidney diseases, such as IgAN, membranous nephropathy, and focal segmental glomerular sclerosis, were excluded. The indications of renal biopsy at our center included continuous proteinuria, rapid decline in renal function, acute kidney injury occurrence, and obvious hematuria. The patient’s demographic and clinical data were retrieved for all patients within one month of renal biopsy. Blood examinations mainly included serum creatinine and serum albumin. Due to most patients having obvious proteinuria and albuminuria not available for all patients, we collected the quantification of total proteinuria instead of albuminuria for our population. The level of estimated glomerular filtration rate (eGFR) was calculated using the Chinese Kidney Disease Epidemiology Collaboration equation.

**II. Model training**

The following subcategories of glomeruli were applied: global glomerulosclerosis (GS), segmental glomerulosclerosis (SS), crescent (C), Kimmelstiel-Wilson lesion (KW, a kind of oval mesangial lesions with an acellular hyaline core and rounded peripherally by mesangial nuclei^2^, the size of acellular hyaline core must be larger than twice the size of the mesangial nucleus), and none of the above lesions (NOA, glomeruli with mild to severe mesangial expansion but without any sclerosis, crescent, or KW lesions). Focal segmental glomerulosclerosis lesion was defined as sclerosis involving a portion of the glomerular tuft, but not involving the whole tuft or just adhesion. Regions excluding glomeruli were defined as negative samples (Neg). To improve the identification performance of KW lesions and minimize disequilibrium from the number disparity of different glomeruli types, patients with the presence of KW lesions were selected as much as possible for model training. This dataset consists of 36,840 fine-annotated glomeruli, including 19,468 NOA, 2,639 SS, 7,171 GS, 1,384 C, and 6,178 KW accompanied by 14,490 Neg samples. This dataset was then split into subsets (train, validation, and test) with a ratio of 3:1:1. As described in our previous study^3^, several CNN algorithms were integrated into the ARPS system comprising 4 procedures: (i) slide preprocessing: tissue extraction and patch overlap-tiling; (ii) glomeruli location; (iii) glomeruli types identification; (iv) glomeruli decomposition: intrinsic cells recognition and mesangial area analysis. In this study, we used the first two steps to finish slide preprocessing and glomeruli location. During the 3rd step, the model developed previously^1^ adopted a two-step strategy to tackle the fine-grained glomerular category problem, firstly picked out the easy-classified categories such as GS glomeruli and Neg images, and further identified the rest such as SS, C, and NOA glomeruli. This two-step strategy is a relatively time-consuming process and is hard to maintain for adding new glomerular lesion types. In this study, we developed an end-to-end efficient neural network based on EfficientNet to identify the glomeruli types that were suitable for DN patients, which utilizes a combination of training-aware neural network search and scaling to jointly optimize model efficiency. This model is trained to identify NOA, SS, GS, C, and KW glomeruli simultaneously. To extract the fine-grained features and capture the non-local information in glomeruli types, we armed the model with multi-head self-attention to solve the long-range dependency problem on glomeruli categories.

After glomeruli were classified as subcategories, we picked out NOA and KW glomeruli in which no obvious lesions affect the mesangial margin. The experienced pathologist labeled along the margin of each mesangium region was blinded to clinical information. For training the mesangial area segmentation model, a total of 894 glomeruli were annotated, with 536 for training, 179 for validation, and 179 for testing. Then these annotated samples were fed into a CNN model. As described in our previous work^3^, a combination of the U-Net variant and marker-controlled watershed segmentation was proved to segment mesangial areas in IgAN patients. For better segmenting mesangial area inside NOA and KW glomeruli among patients with DN, a novel CNN model based on U-Net was designed to get accurate segmentation through training with fine-annotated mesangial area from DN glomeruli, as U-Net architecture was the most powerful CNN algorithm for tissue segmentation^4^.

The ARPS system constructed in our prior study has employed V-Net architecture to predict glomerular intrinsic cells accurately in patients with IgAN^3^. Glomeruli in DN could have more severe mesangial expansion or the presence of KW lesions or concomitant severe podocyte depletion compared to IgAN. Here, we verified the performance of this well-trained V-Net architecture in identifying intrinsic cells in patients with DN. NOA and KW glomeruli were selected randomly from the enrolled patients. After having fed these glomeruli into the V-Net architecture, the identification result of each cell inside the glomeruli was then verified by the pathologist.

1. Tervaert TW, Mooyaart AL, Amann K, et al. Pathologic classification of diabetic nephropathy. *J Am Soc Nephrol*. 2010;21:556-563.
2. Stout LC, Kumar S, Whorton EB. Focal mesangiolysis and the pathogenesis of the Kimmelstiel-Wilson nodule. *Hum Pathol*. 1993;24:77-89. doi: 10.1016/0046-8177(93)90066-p

3. Zeng C, Nan Y, Xu F, et al. Identification of glomerular lesions and intrinsic glomerular cell types in kidney diseases via deep learning. *J Pathol*. 2020;252:53-64. doi: 10.1002/path.5491

4. Ronneberger. O, Fischer. P, Brox. T. U-Net: Convolutional Networks for Biomedical Image Segmentation. *arXiv*. 2015:1505.04597. doi:

**III. Model evaluation**

For evaluating the performance of the classification model on categorizing glomeruli types or predicting glomerular intrinsic cells, a confusion matrix from testing data was established to present the prediction of our CNN model relative to the ground truth (assessed by pathologists) in patients with DN. Since only light microscopy, infiltrate cells and endothelial cells cannot be completely distinguished. Common evaluation indices calculated from the confusion matrix comprise accuracy, specificity, precision, and recall as follows. F1-score was a comprehensive index simultaneously considering accuracy and recall, which was the most common index to evaluate the performance of classification models.

**Confusion matrix of classification model**

| Confusion matrix | | Ground truth | |
| --- | --- | --- | --- |
|  |  | Positive | Negative |
| Prediction | Positive | TP (True positive) | FP (False positive) |
|  | Negative | FN (False negative) | TN (True negative) |

**The calculating methods of common evaluation indices in the classification model**

| Indices | Calculating formula |
| --- | --- |
| Accuracy | $=\frac{TP+TN}{TP+TN+FP+FN}$ |
| Specificity | $=\frac{\mathrm{TN}}{TN+FP}$ |
| Precision | $=\frac{\mathrm{TP}}{TP+FP}$ |
| Recall | $=\frac{\mathrm{TP}}{TP+FN}$ |
| F1-score | $=\frac{2*precision*recall}{precision+recall}$ |

For the segmentation model, the Dice index was used to evaluate the performance of the model for segmenting mesangial area.

$$Dice=\frac{2\left| P\bigcap\mathrm{GT} \right|}{\left| P \right|+\left| \mathrm{GT} \right|}$$

where GT represents the regions of the ground truth given by the pathologist, and P indicates the segmenting regions by the trained model.

**IV. Calculating methods for** **intraglomerular features**

Given that the recommended pathologic classification was based on a tissue cross-section in clinical practice, we also quantified intraglomerular features in a cross-section in this study. The glomeruli with a total number of intrinsic cells less than 50 were excluded from analyses to diminish the variance from non-midsection glomeruli. The mesangial area fraction (mesangial area/glomerular area ratio) was defined to reflect the degrees of mesangial expansion relative to glomerular size. Mesangial extracellular matrix and mesangial cytoplasm could not be distinguished under PAS staining. To evaluate the degrees of mesangial matrix accumulation not affected by mesangial cell proliferation, the mesangial area/mesangial cell ratio in a glomerular section was also calculated. The cell density (intrinsic cell number/glomerular area ratio) and cell ratio (intrinsic cell number/total intrinsic cell ratio) were defined to reflect the number change of intrinsic cells relative to glomerular size and total intrinsic cell numbers, respectively. The detailed calculating methods of intraglomerular features for each patient are described as follows:

$$Intrinsic cell density =\frac{Average (intrinsic cell numbers per glomerular section)}{Average (glomerular area)}$$

$$Intrinsic cell ratio=\frac{\sum(\frac{intrinsic cell number per glomerular section}{total intrinsic cell numbers per glomerular section})}{glomeruli number}$$

$Mesangial area fraction=\frac{Average(mesangial area per glomerular section)}{Average(glomerular area)}$

$$Mesangial area/mesangial cell ratio=\frac{\sum(\frac{mesangial area per glomerular section}{mesangial cell numbers per glomerular section})}{glomeruli number}$$

**Supplementary results**

**Table S1. Patients’ characteristics in different subsets**

| Variables | Glomeruli types model  (n=398) | Mesangial area model  (n=119) | Internal application  (n=226) | External application  (n=114) |
| --- | --- | --- | --- | --- |
| Male, n (%) | 278 (69.8) | 92 (77.3) | 175 (77.4) | 92 (80.7) |
| Age, years | 50 (45, 57) | 50 (42, 56) | 51 (44, 57) | 52 (45, 57) |
| BMI, kg/m^2^ | 24.5 (22.5, 27.1) | 25.0 (22.6, 27.4) | 24.7 (22.6, 27.7) | 25.9 (23.4, 28.1) |
| HBP, n (%) | 322 (81.5) | 96 (83.5) | 168 (76.0) | 85 (77.3) |
| Serum creatinine, mg/dl | 1.52 (1.08, 2.05) | 1.28 (0.97, 1.79) | 1.35 (1.01, 1.89) | 1.27 (0.95, 1.62) |
| Serum albumin, g/L | 35.1 (29.8, 39.0) | 36.7 (32.0, 43.0) | 35.8 (31.1, 41.2) | 39.8 (33.1, 44.1) |
| Urine protein, g/24 hour | 3.6 (1.8, 6.2) | 3.0 (1.5, 4.8) | 3.2 (1.4, 6.1) | 2.2 (1.1, 4.5) |

Abbreviations: BMI, body mass index; HBP, high blood pressure.

Note: Continuous variables were presented as median (interquartile range). Categorical variables were presented as numbers (percentages).

**Table S2. Confusion matrix of the classification model on predicting different glomeruli types**

| Confusion matrix | | Ground truth | | | | | | |
| --- | --- | --- | --- | --- | --- | --- | --- | --- |
|  |  | C | GS | KW | NOA | SS | Neg1 | Total |
| Prediction | C | 374 | 11 | 2 | 44 | 89 | 0 | 520 |
|  | GS | 12 | 998 | 23 | 1 | 29 | 70 | 1133 |
|  | KW | 1 | 0 | 1186 | 18 | 3 | 4 | 1212 |
|  | NOA | 18 | 0 | 56 | 3582 | 138 | 126 | 3920 |
|  | SS | 38 | 7 | 9 | 115 | 380 | 0 | 549 |
|  | Neg1 | 0 | 1 | 1 | 3 | 0 | 2465 | 2470 |
|  | Total | 443 | 1017 | 1277 | 3763 | 639 | 2665 | 9804 |

Note: GS, global glomerulosclerosis; SS, segmental glomerulosclerosis; C, crescent; KW, Kimmelstiel–Wilson lesions; NOA, none of the above lesions; Neg1, regions excluding glomeruli were defined as negative samples.

**Table S3. Confusion matrix of the classification model** **on predicting three intrinsic cells**

| Confusion matrix | | Ground truth | | | | |
| --- | --- | --- | --- | --- | --- | --- |
|  |  | M | E | P | Neg2 | Total |
| Prediction | M | 4975 | 326 | 33 | 2 | 5336 |
|  | E | 176 | 3920 | 63 | 15 | 4174 |
|  | P | 54 | 66 | 1333 | 45 | 1498 |
|  | Neg2 | 165 | 273 | 252 | 1374 | 2064 |
|  | Total | 5370 | 4585 | 1681 | 1436 | 13072 |

Note: M, mesangial cells; E, endothelial cells; P, podocytes; Neg2, cells inside Bowman capsule excluding the above three glomerular intrinsic cells (e.g., parietal epithelial cells, inflammatory cells).

**Table S4. The level of intraglomerular features in different pathologists-based classes (n=340)**

| Classes | The average area per glomerular section | | | |  | Average cell numbers per glomerular section | | | | |
| --- | --- | --- | --- | --- | --- | --- | --- | --- | --- | --- |
|  | Glomeruli  (×10^3^ μm^2^) | Mesangium  (×10^3^ μm^2^) | Mesangial area/mesangial cell ratio (μm^2^) | Mesangial area fraction |  | Total cells | Mesangial cells | Endothelial cells | | Podocytes |
| I  (n=17) | 25.5 (22.7, 26.6) | 3.4 (3.3, 3.9) | 87.3 (71.6, 97.9) | 0.14 (0.13, 0.16) |  | 109.0 (103.8, 121.6) | 44.1 (35.1, 48.7) | | 46.2 (42.0, 49.8) | 21.2 (19.2, 26.4) |
| IIa (n=49) | 28.6 (24.4, 31.5) | 4.9 (4.3, 5.9) | 115.7 (103.6, 128.4) | 0.18 (0.16, 0.21) |  | 108.8 (101.1, 120.7) | 44.6 (36.5, 51.0) | | 46.3 (42.4, 52.5) | 18.6 (17.7, 20.9) |
| IIb (n=27) | 27.8 (26.0, 32.9) | 8.0 (5.8, 9.4) | 138.3 (123.0, 161.7) | 0.26 (0.23, 0.29) |  | 120.7 (107.9, 137.1) | 57.5 (46.0, 64.4) | | 49.9 (43.1, 55.0) | 18.0 (15.1, 20.9) |
| III (n=174) | 28.1 (25.3, 31.7) | 9.5 (8.0, 11.7) | 149.4 (133.7, 169.8) | 0.34 (0.31, 0.39) |  | 126.6 (114.5, 139.4) | 62.0 (54.3, 74.0) | | 47.4 (42.1, 52.0) | 15.5 (13.1, 17.5) |
| IV (n=73) | 28.3 (24.7, 31.7) | 9.1 (7.0, 11.3) | 153.6 (126.1, 175.1) | 0.32 (0.27, 0.38) |  | 122.9 (107.8, 137.0) | 60.4 (48.5, 72.1) | | 46.0 (39.1, 53.8) | 14.8 (12.5, 16.4) |
| *P* value | 0.068 | <0.001 | <0.001 | <0.001 |  | <0.001 | <0.001 | | 0.431 | <0.001 |

Note: Data were presented as median (interquartile range: IQR), and inter-group comparisons were performed by the Kruskal-Wallis H test.

**Table S5. The level of intrinsic cell density in different pathologists-based classes (n=340)**

| Class | Mesangial cells density (n/10^3^ μm^2^) | Endothelial cells density  (n/10^3^ μm^2^) | Podocytes density  (n/10^3^ μm^2^) |
| --- | --- | --- | --- |
| I | 1.63 (1.49, 1.92) | 1.84 (1.65, 2.04) | 0.92 (0.72, 1.03) |
| IIa | 1.55 (1.35, 1.75) | 1.66 (1.53, 1.82) | 0.67 (0.55, 0.79) |
| IIb | 1.80 (1.68, 2.11) | 1.76 (1.61, 1.89) | 0.60 (0.51, 0.75) |
| III | 2.21 (1.94, 2.60) | 1.65 (1.53, 1.79) | 0.54 (0.46, 0.63) |
| IV | 2.09 (1.85, 2.42) | 1.61 (1.42, 1.76) | 0.51 (0.42, 0.59) |
| *P* value | <0.001 | 0.003 | <0.001 |

Note: Data were presented as median (interquartile range: IQR), and inter-group comparisons were performed by the Kruskal-Wallis H test.

**Table S6. The comparison of intrinsic cell ratio between NOA and KW glomeruli**

| Glomeruli types | Total cells numbers | Mesangial cells ratio | Endothelial cells ratio | Podocytes ratio |
| --- | --- | --- | --- | --- |
| NOA (n=8181) | 114 (86, 145) | 0.44 (0.38, 0.51) | 0.40 (0.35, 0.45) | 0.15 (0.10, 0.20) |
| KW (n=3007) | 138 (102, 175) | 0.54 (0.47, 0.61) | 0.35 (0.30, 0.40) | 0.10 (0.07, 0.14) |
| *P* value | <0.001 | <0.001 | <0.001 | <0.001 |

Note: Data were presented as median (interquartile range: IQR), and inter-group comparisons were performed by the Mann-Whitney U test.

**Table S7. ROC curve analysis of intraglomerular morphological features on distinguishing early classes.**

| **Variables** | **AUC** | ***P* value** | **95% CI** | **Cutoff value** | **Accuracy** | **Specificity** |
| --- | --- | --- | --- | --- | --- | --- |
| **Class IIa from class I** |  |  |  |  |  |  |
| Average mesangial area | 0.815 | 0.017 | 0.658-0.971 | 4336.2 | 0.741 | 0.833 |
| Mesangial area/mesangial cell ratio | 0.951 | 0.001 | 0.879-1.000 | 105.4 | 0.852 | 1.000 |
| Mesangial area fraction | 0.809 | 0.020 | 0.642-0.975 | 0.157 | 0.815 | 0.667 |
| **Class IIb from class IIa** |  |  |  |  |  |  |
| Average mesangial area | 0.861 | <0.001 | 0.749-0.972 | 5959.5 | 0.765 | 0.815 |
| Average mesangial cell number | 0.704 | 0.024 | 0.546-0.862 | 45.84 | 0.765 | 0.630 |
| Mesangial area/mesangial cell ratio | 0.824 | <0.001 | 0.702-0.945 | 118.6 | 0.941 | 0.593 |
| Mesangial area fraction | 0.948 | <0.001 | 0.887-1.0 | 0.207 | 0.941 | 0.852 |
| Mesangial cell density | 0.810 | 0.001 | 0.668-0.953 | 0.002 | 0.706 | 0.889 |

Note: AUC, the area under the ROC curve; CI, confidence interval. The cutoff value is derived from the point with the maximum accuracy plus specificity.

**Table S8.** **Spearman correlation of clinical prognostic indicators with CNN-based or pathologists-based classes in the external application subset (Model 1)**

| Indicators | Proteinuria | | | |  | eGFR | | | | | |
| --- | --- | --- | --- | --- | --- | --- | --- | --- | --- | --- | --- |
|  | Proteinuria  at biopsy,  g/24h  (n=113) | | Time-average proteinuria, g/24h  (n=91) | |  | eGFR at biopsy, ml/min/1.73 m^2^  (n=114) | | eGFR slope, ml/min/1.73 m^2^/year  (n=93) | | Event of ESRD  (n=93) | |
| **Spearman correlation (*r*, *P value*)** | *r* | *P value* | *r* | *P value* |  | *r* | *P value* | *r* | *P value* | *r* | *P value* |
| Pathologists-based classes | 0.562 | <0.001 | 0.577 | <0.001 |  | -0.468 | <0.001 | -0.517 | <0.001 | 0.306 | 0.003 |
| CNN-based classes | 0.582 | <0.001 | 0.613 | <0.001 |  | -0.480 | <0.001 | -0.559 | <0.001 | 0.245 | 0.018 |
| **Z-scores (Z, *P value*)** | Z | *P value* | Z | *P value* |  | Z | *P value* | Z | *P value* | Z | *P value* |
| CNN vs pathologists | -0.458 | 0.647 | -0.755 | 0.450 |  | 0.255 | 0.799 | -0.845 | 0.398 | 1.062 | 0.289 |

Note: CNN-based classes derived from Model 1 (combined with average mesangial area). The Spearman r coefficient of CNN-based classes with pathologists-based classes was 0.838.

**Table S9. Spearman correlation of clinical prognostic indicators** **with CNN-based or pathologists-based classes in the internal application subset (Model 2)**

| Indicators | Proteinuria | | | |  | eGFR | | | | | |
| --- | --- | --- | --- | --- | --- | --- | --- | --- | --- | --- | --- |
|  | Proteinuria  at biopsy, g/24h  (n=226) | | Time-average proteinuria, g/24h  (n=157) | |  | eGFR at biopsy, ml/min/1.73 m^2^  (n=226) | | eGFR slope, ml/min/1.73 m^2^/year  (n=157) | | Event of ESRD  (n=157) | |
| **Spearman correlation (*r*, *P value*)** | *r* | *P value* | *r* | *P value* |  | *r* | *P value* | *r* | *P value* | *r* | *P value* |
| Pathologists-based classes | 0.327 | <0.001 | 0.447 | <0.001 |  | -0.443 | <0.001 | -0.363 | <0.001 | 0.250 | 0.002 |
| CNN-based classes | 0.362 | <0.001 | 0.386 | <0.001 |  | -0.369 | <0.001 | -0.316 | <0.001 | 0.231 | 0.004 |
| **Z-scores (Z, *P value*)** | Z | *P value* | Z | *P value* |  | Z | *P value* | Z | *P value* | Z | *P value* |
| CNN vs pathologists | -0.740 | 0.460 | 1.113 | 0.266 |  | -1.615 | 0.106 | 0.824 | 0.410 | 0.321 | 0.748 |

Note: CNN-based classes derived from Model 2 (combined with mesangial area/mesangial cell ratio). The Spearman r coefficient of CNN-based classes with pathologists-based classes was 0.711.

**Table S10.** **Spearman correlation of clinical prognostic indicators with CNN-based or pathologists-based classes in the external application subset (Model 2)**

| Indicators | Proteinuria | | | |  | eGFR | | | | | |
| --- | --- | --- | --- | --- | --- | --- | --- | --- | --- | --- | --- |
|  | Proteinuria  at biopsy,  g/24h  (n=113) | | Time-average proteinuria, g/24h  (n=91) | |  | eGFR at biopsy, ml/min/1.73 m^2^  (n=114) | | eGFR slope, ml/min/1.73 m^2^/year  (n=93) | | Event of ESRD  (n=93) | |
| **Spearman correlation** **(*r*, *P value*)** | *r* | *P value* | *r* | *P value* |  | *r* | *P value* | *r* | *P value* | *r* | *P value* |
| Pathologists-based classes | 0.562 | <0.001 | 0.577 | <0.001 |  | -0.468 | <0.001 | -0.517 | <0.001 | 0.306 | 0.003 |
| CNN-based classes | 0.566 | <0.001 | 0.611 | <0.001 |  | -0.477 | <0.001 | -0.555 | <0.001 | 0.240 | 0.021 |
| **Z-scores (Z, *P value*)** | Z | *P value* | Z | *P value* |  | Z | *P value* | Z | *P value* | Z | *P value* |
| CNN vs pathologists | -0.088 | 0.930 | -0.689 | 0.491 |  | 0.185 | 0.854 | 0.737 | 0.461 | 1.108 | 0.0.268 |

Note: CNN-based classes derived from Model 2 (combined with mesangial area/mesangial cell ratio). The Spearman r coefficient of CNN-based classes with pathologists-based classes was 0.826.

**Table S11. Spearman correlation of clinical prognostic indicators with CNN-based or pathologists-based classes in the internal application subset (Model 3)**

| Indicators | Proteinuria | | | |  | eGFR | | | | | |
| --- | --- | --- | --- | --- | --- | --- | --- | --- | --- | --- | --- |
|  | Proteinuria  at biopsy,  g/24h  (n=226) | | Time-average proteinuria, g/24h  (n=157) | |  | eGFR at biopsy, ml/min/1.73 m^2^  (n=226) | | eGFR slope, ml/min/1.73 m^2^/year  (n=157) | | Event of ESRD  (n=157) | |
| **Spearman correlation (*r*, *P value*)** | *r* | *P value* | *r* | *P value* |  | *r* | *P value* | *r* | *P value* | *r* | *P value* |
| Pathologists-based classes | 0.327 | <0.001 | 0.447 | <0.001 |  | -0.443 | <0.001 | -0.363 | <0.001 | 0.250 | 0.002 |
| CNN-based classes | 0.372 | <0.001 | 0.390 | <0.001 |  | -0.371 | <0.001 | -0.322 | <0.001 | 0.232 | 0.003 |
| **Z-scores (Z, *P value*)** | Z | *P value* | Z | *P value* |  | Z | *P value* | Z | *P value* | Z | *P value* |
| CNN vs pathologists | -0.966 | 0.334 | 1.055 | 0.291 |  | -1.594 | 0.111 | -0.729 | 0.466 | 0.308 | 0.758 |

Note: CNN-based classes derived from Model 3 (combined with mesangial area fraction). The Spearman r coefficient of CNN-based classes with pathologists-based classes was 0.719.

**Table S12. Spearman correlation of** **clinical prognostic indicators with CNN-based or pathologists-based classes in the external application subset (Model 3)**

| Indicators | Proteinuria | | | |  | eGFR | | | | | |
| --- | --- | --- | --- | --- | --- | --- | --- | --- | --- | --- | --- |
|  | Proteinuria  at biopsy,  g/24h  (n=113) | | Time-average proteinuria, g/24h  (n=91) | |  | eGFR at biopsy, ml/min/1.73 m^2^  (n=114) | | eGFR slope, ml/min/1.73 m^2^/year  (n=93) | | Event of ESRD  (n=93) | |
| **Spearman correlation** **(*r*, *P value*)** | *r* | *P value* | *r* | *P value* |  | *r* | *P value* | *r* | *P value* | *r* | *P value* |
| Pathologists-based classes | 0.562 | <0.001 | 0.577 | <0.001 |  | -0.468 | <0.001 | -0.517 | <0.001 | 0.306 | 0.003 |
| CNN-based classes | 0.572 | <0.001 | -0.603 | <0.001 |  | -0.490 | <0.001 | -0.554 | <0.001 | 0.245 | 0.018 |
| **Z-scores** **(Z, *P value*)** | Z | *P value* | Z | *P value* |  | Z | *P value* | Z | *P value* | Z | *P value* |
| CNN vs pathologists | -0.227 | 0.821 | -0.540 | 0.590 |  | 0.467 | 0.641 | 0.738 | 0.460 | 1.055 | 0.291 |

Note: CNN-based classes derived from Model 3 (combined with mesangial area fraction). The Spearman r coefficient of CNN-based classes with pathologists-based classes was 0.836.

Figure S1


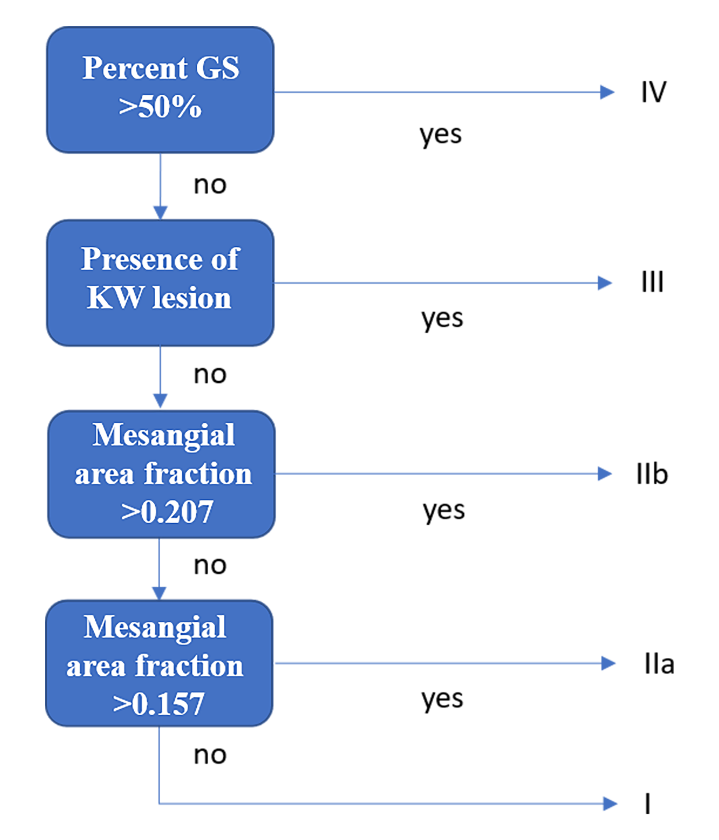

Supplement: Supplementary file 1 — Additional file 1. Supplementary Material [file 12967_2024_5221_MOESM1_ESM.docx]
